# Key Factors in Helpfulness and Use of the SAFE Intervention for Women Experiencing Intimate Partner Violence and Abuse: Qualitative Outcomes From a Randomized Controlled Trial and Process Evaluation

**DOI:** 10.2196/42647

**Published:** 2023-08-21

**Authors:** Nicole E van Gelder, Suzanne A Ligthart, Karin A W L van Rosmalen-Nooijens, Judith B Prins, Sabine Oertelt-Prigione

**Affiliations:** 1 Department of Primary and Community Care Research Institute for Medical Innovation Radboud University Medical Center Nijmegen Netherlands; 2 Department of Medical Psychology Research Institute for Medical Innovation Radboud University Medical Center Nijmegen Netherlands; 3 Arbeitsgruppe 10 Geschlechtersensible Medizin Medical Faculty Ostwestfalen-Lippe Bielefeld University Bielefeld Germany

**Keywords:** intimate partner violence and abuse, domestic violence and abuse, eHealth, web based, web-based intervention, help seeking, interview, qualitative, randomized controlled trial, process evaluation

## Abstract

**Background:**

Many women experience at least one type of intimate partner violence and abuse (IPVA), and although various support options are available, we still know relatively little about web-based interventions for IPVA survivors. We conducted a qualitative evaluation of the SAFE eHealth intervention for women experiencing IPVA in the Netherlands, complementing the quantitative evaluation of self-efficacy, depression, anxiety, and multiple feasibility aspects.

**Objective:**

This study assessed users’ experiences and what, according to them, were useful and helpful aspects of the intervention.

**Methods:**

The intervention consisted of modules with information on relationships and IPVA, help options, physical and mental health, and social support. It also contained interactive elements such as exercises, stories from survivors, a chat, and a forum. A randomized controlled trial was conducted with an intervention arm receiving the complete version of the intervention and a control arm receiving only a static version with the modules on relationships and IPVA and help options. We gathered data through open questions from surveys (for both study arms; n=65) and semistructured interviews (for the intervention study arm; n=10), all conducted on the web, during the randomized controlled trial and process evaluation. Interview data were coded following the principles of open thematic coding, and all qualitative data were analyzed using qualitative content analysis.

**Results:**

Overall, most users positively rated the intervention regarding safety, content, and suiting their needs, especially participants from the intervention study arm. The intervention was helpful in the domains of acknowledgment, awareness, and support. However, participants also identified points for improvement: the availability of a simplified version for acute situations; more attention for survivors in the aftermath of ending an abusive relationship; and more information on certain topics, such as technological IPVA, support for children, and legal affairs. Furthermore, although participants expressed a prominent need for interactive contact options such as a chat or forum, the intervention study arm (the only group that had these features at their disposal) mainly used them in a passive way—reading instead of actively joining the conversation. The participants provided various reasons for this passive use.

**Conclusions:**

The positive outcomes of this study are similar to those of other web-based interventions for IPVA survivors, and specific points for improvement were identified. The availability of interactive elements seems to be of added value even when they are used passively. This study provides in-depth insight into the experiences of female IPVA survivors with the SAFE eHealth intervention and makes suggestions for improvements to SAFE and comparable web-based interventions for IPVA as well as inspiring future research. Furthermore, this study shows the importance of a varied assessment of an intervention’s effectiveness to understand the real-world impact on its users.

**Trial Registration:**

Netherlands Trial Register NTR7313; https://tinyurl.com/3t7vwswz

## Introduction

### Background

Approximately 30% of women worldwide experience physical or sexual violence by a current partner or ex-partner during their lifetime [1-3]. Intimate partner violence and abuse (IPVA) occur in various forms: physical, psychological, sexual, and economic [4]. Although many women encounter this type of violence, it is still difficult for survivors to seek help. Feelings of fear, shame, and guilt discourage them from reaching out to their informal network or professional help. Other obstacles such as a lack of knowledge of IPVA and possible help options, logistical issues, and the fear of losing their children negatively influence the help-seeking process as well [5,6].

Especially in light of the COVID-19 pandemic [7], with a high rise of IPVA, web-based tools have become more important [8-10]. In the Netherlands, several domestic violence and abuse (DVA) organizations have started to offer this type of support, for example, via an anonymous chat [11]. Web-based means have the potential to lower the threshold for disclosing IPVA and seeking help. Although web-based interventions do not aim to and cannot replace regular (face-to-face) help, they do have the ability to reach survivors who otherwise might not be reached. Overall, survivors feel that a web-based medium such as an app or website is helpful and has advantages in accessibility, anonymity, privacy, trust, nonjudgmental tone, awareness, help seeking, social support, and autonomy [12-20]. However, web-based support will not be suitable for everyone and, although in the Netherlands most of the general population has internet access and many have adequate digital skills [21,22], not everyone will have the opportunity to use a web-based intervention [23-25].

This study focuses on the qualitative evaluation of the Dutch eHealth intervention SAFE [26] for women who experience IPVA. The intervention is inspired by a Dutch (Feel the ViBe) and an Australian (I-DECIDE) web-based intervention for DVA and IPVA survivors, respectively [27,28]. SAFE is based on scientific insights and the views of survivors and professionals [18] and aims to improve self-efficacy, mental health, and awareness and encourage women to seek help. The evaluation consisted of a randomized controlled trial (RCT), a process evaluation, and an open feasibility study. The study protocol and the outcomes of the quantitative evaluation have been described elsewhere [29,30]. The RCT and process evaluation participants were divided into 2 groups, with the intervention study arm receiving the complete version of the intervention and the control study arm receiving a static version with only the essential information on IPVA and help options (this is further explained in the *Methods* section and Textbox 1). In short, the quantitative evaluation showed no significant differences between the study arms in self-efficacy and mental health outcomes. However, it did show positive outcomes for feasibility aspects, such as demand and acceptability. Furthermore, both groups significantly improved over time in anxiety and fear of their partner, and both groups were in general satisfied with SAFE, with the intervention group being significantly more positive [30].

**SAFE intervention: modules and functionalities during the randomized controlled trial. Text in italics indicates availability to the control and intervention arms; text not in italics indicates availability only to the intervention arm.**

**Module: My situation**

- *Information on intimate partner violence and abuse (IPVA)*
- *Information on healthy and unhealthy relationships*
- *Information on the impact of IPVA on children*

**Module: Help**

- *Information on various help options*
- *Information on safety (eg, in preparing to leave an abusive partner)*
- *Help database with help options, including filters for type of help and region*

**Module: My health**

- Information on physical health (issues)
- Information on mental health (issues)
- Information on coping strategies and stress reduction

**Module: My environment**

- Information on social support
- Information on disclosing IPVA
- Information on contact options

**Contact**

- *Links to contact options with fellow survivors*
- *Option to contact the community managers*
- Chat, forum, and diary

**About SAFE**

- *Information on the SAFE research project (including patient information letter and stakeholders who provided input)*
- *Information on safety measures*
- *Information on the community managers*

**Additional functionalities (throughout the intervention when applicable)**

- Exercises for creating awareness and to stimulate reflection on their situation and help-seeking process
- Short videos by female survivors of IPVA and by professionals
- Stories and quotes from female survivors of IPVA
- *Tips for books, films, and activities*

### Objectives

This paper describes the outcomes of the qualitative evaluation, which consisted of the users’ experiences with and views on the SAFE eHealth intervention. It provides in-depth insight into what female IPVA survivors need in web-based support and how, according to them, it can benefit them.

## Methods

### Ethics Approval

This study was conducted in compliance with the Declaration of Helsinki, and all study components were approved by the Medical Ethics Committee of Arnhem-Nijmegen (NL68268.091.18; dossier 2018-5009).

### Informed Consent and Participation

All participants in the RCT received a digital information letter and provided consent digitally by checking a box. After a mandatory waiting period of 24 hours to ensure sufficient time for making the decision to participate, they were asked to digitally provide consent a second time before they were enrolled. All participants remained anonymous; they did not have to use their real names anywhere. The women who completed all questionnaires received a generic digital gift card for €20 (US $22.42). The protocol paper of this study elaborates on the ethical considerations [29].

RCT participants were asked if we were allowed to contact them via email for follow-up research. Only the women who consented to this were invited to participate in a web-based interview. Women who responded positively digitally received an information letter and signed an informed consent form. The participants could remain anonymous as they did not have to use their real names, and during the web-based interview, they could leave the camera off. All personal identifiers were removed from the quotes in this paper. Interview participants received a generic digital gift card for €20 (US $22.42) after the interview was conducted. We refer to the protocol paper for further details on the ethical considerations [29].

### Study Design

This study consisted of an RCT and a qualitative process evaluation. For the RCT, participants were divided into 2 age groups (18-30 years and 31-50 years) to ensure an even age division across groups, and they were randomly assigned to the control or intervention group. The control group had access to a static version of the intervention containing modules with essential information on IPVA and help options, including a help database. The intervention group received the complete intervention, which comprised all the elements in the static version and also included extra modules on (mental) health and social support and interactive components such as short movies from IPVA survivors, a chat, and a forum (Textbox 1). The intervention did not have a predetermined order that participants needed to follow; they could choose how and when to use certain components of the intervention according to their personal situation and needs. For a more elaborate description of the intervention and study components, we refer to the earlier published protocol [29] and the quantitative evaluation [30]. The qualitative parts of the RCT and process evaluation are discussed in this study, and they consist of data from open questions, the chat and forum, and interviews.

### Recruitment, Data Acquisition, and Measures

For the RCT, recruitment mainly took place on the web through social media and presence in the (web-based) media. Women aged between 18 and 50 years who self-identified as IPVA survivors were included between April 1, 2019, and October 1, 2020. They filled out a General Characteristics Questionnaire (GCQ) at 3, 6, and 12 months and a Web Evaluation Questionnaire (WEQ) at 1, 3, and 6 months. The WEQ contained open questions on evaluating the intervention, such as “What do you think is positive or helpful, and what are points of improvement?” and “Do you feel safe on the website? Please explain why (not).” The data from the chat and forum were collected between April 1, 2019, and April 1, 2021.

For the process evaluation, participants from the RCT intervention group were recruited. This evaluation consisted of semistructured interviews that were conducted between February 2, 2021, and March 19, 2021. All interviewees registered for the SAFE intervention between 6 and 22 months before the interview (mean 13, SD 5.6 months). The interview guide (Multimedia Appendix 1) contained questions on the women’s personal experiences with IPVA and their experiences with the intervention.

### Study Procedures

Participants enrolled in the study digitally received an information letter and provided consent before participating. They also received the aforementioned questionnaires at various time points. Regardless of whether the participants filled out these questionnaires, they retained access to the intervention also when the follow-up period was completed. During the RCT period, we automatically gathered data from the chat and forum.

As stated before, participants from the RCT intervention group who agreed to being approached for additional research were contacted via email with an invitation to participate in the interview study. They received a digital information letter and signed an informed consent form. The interviews were conducted by a female researcher (NEvG) with previous interviewing experience with IPVA survivors and a background in social sciences. The audio from all interviews was recorded, transcribed verbatim, and anonymized.

### Analysis

The data from the chat and forum were summarized and analyzed through qualitative content analysis [31], highlighting the themes and participants’ questions that were discussed. The data from the open questions stemming from the GCQ and WEQ were summarized and analyzed through qualitative content analysis as well [31]. Quantitative data from the WEQ were collected at multiple time points (question on grading the website after 1 month: n=28; question on grading the website after 3 months: n=15; question on grading the website after 6 months: n=13). To avoid overlap and, as a result, a biased image of the users’ experiences, only the most recent outcomes were included in the analysis for participants who filled out multiple follow-up surveys as this best reflects their concluding, overall perception of the intervention after a longer period of use [30]. All qualitative data from the GCQ (question on experience with SAFE after 3 months: n=35; question on experience with SAFE after 6 months: n=26; question on experience with SAFE after 12 months: n=6) and the WEQ were included in this study’s analysis to gather all reflections and feedback on the intervention. For these qualitative outcomes of the surveys, a comparison between the control and intervention arms was made if applicable, and attention was paid to both the similarities and differences in experiences.

All interview data were coded and analyzed using ATLAS.ti (version 8.4.20; ATLAS.ti Scientific Software Development GmbH) [32].

Coding was conducted by 1 researcher (NEvG) and 2 research assistants (Arrantxa Groot and Lieke Gommans) independently following the principles of open and thematic coding [33]. After several discussion rounds, consensus was reached on the codes, categories, themes, and the final codebook with which all interviews were reread to ensure that all data were included and correctly coded. The data were analyzed using qualitative content analysis [31]. Text segments were used to illustrate the outcomes.

The data from various sources (questionnaires, interviews, chat, and forum) were analyzed separately and subsequently combined into 4 main themes that were derived from the data across the sources: use and ease of use, safety, level of satisfaction and helpfulness, and points of improvement.

## Results

### Participant Characteristics

The survey group consisted of 65 women from the control (37/99, 37%) and intervention (28/99, 28%) study arms who filled out one or more follow-up GCQs and WEQs (Table 1). This group had a mean age of 38 years when they registered for SAFE (month 0), and 88% (57/65) were born in the Netherlands. Approximately 91% (59/65) identified (partially) as Dutch, 58% (38/65) were highly educated, 63% (41/65) were employed, 91% (59/65) identified as heterosexual, and 69% (45/65) of the participants had children.

In the interview group, initially 12 women wanted to participate, but 2 (17%) changed their minds. Therefore, this study sample consisted of 10 women—thus still reaching code saturation [34]—from the intervention study arm with a mean age of 43 years at month 0 (Table 2); 80% (8/10) of them were also part of the aforementioned survey group. Similar to the survey group, most women were born in the Netherlands (9/10, 90%), and 70% (7/10) of them identified solely as Dutch. The group was relatively highly educated (7/10, 70%), half (5/10, 50%) of the women were employed, and most of them were heterosexual and had children (8/10, 80% in both cases). In the survey and interview group together, most (40/67, 60%) experienced the last IPVA incident in the week of registering for SAFE (month 0).

**Table 1:** Demographics of the survey group at baseline (month 0; n=65^a^).

Demographics			Values
**Study arm, n (%)**			
	Control	37 (57)	
	Intervention	28 (43)	
**Age (years), mean (SD)**			38 (8.3)
	18-30, n (%)	15 (23)	
	31-50, n (%)	50 (77)	
**Sexual orientation, n (%)**			
	Heterosexual	59 (91)	
	Lesbian	2 (3)	
	Bisexual	2 (3)	
	Rather not say	2 (3)	
**Country of birth, n (%)**			
	The Netherlands	57 (88)	
	Belgium	3 (5)	
	Italy	1 (2)	
	Luxembourg	1 (2)	
	Russia	1 (2)	
	Suriname	1 (2)	
	United States	1 (2)	
**Cultural identification^b^, n (%)**			
	Dutch	59 (91)	
	Belgian	3 (5)	
	German	1 (2)	
	Indonesian	2 (3)	
	Moroccan	1 (2)	
	Suriname	1 (2)	
	Turkish	1 (2)	
	Other^c^	6 (9)	
**Religious identification, n (%)**			
	None or atheism	42 (65)	
	Christianity	9 (14)	
	Islam	4 (6)	
	Other^d^	10 (15)	
**Educational level, n (%)**			
	Primary school	1 (2)	
	Secondary school	6 (9)	
	Vocational education	20 (31)	
	Higher vocational education	19 (29)	
	University	16 (25)	
	Postdoctoral	3 (5)	
**Paid employment, n (%)**			
	Yes	41 (63)	
	No	24 (37)	
**Children, n (%)**			
	Yes	45 (69)	
	No	20 (31)	
**Living situation, n (%)**			
	Alone	11 (17)	
	With current partner or ex-partner	11 (17)	
	With children	20 (31)	
	With current partner or ex-partner and children	20 (31)	
	With parents	3 (5)	
**Type of IPVA^e,f^, n (%)**			
	Psychological	63 (97)	
	Physical	46 (71)	
	Sexual	24 (37)	
	Economic	29 (45)	

^a^The survey group consisted of participants who filled out the General Characteristics Questionnaire open question on their experience with SAFE or the Web Evaluation Questionnaire at least once.

^b^Participants could identify with multiple cultural backgrounds.

^c^Other: American, French, Italian, Luxembourgish, Russian, and Western European.

^d^Other: agnostic, partially Hindu, orthodox and Jewish, and spiritual.

^e^Participants could tick multiple boxes.

^f^IPVA: intimate partner violence and abuse.

**Table 2:** Demographics of the interview group at baseline (month 0; n=10).

Participant number	Age (years)	Sexual orientation	Country of birth	Cultural ID	Religious ID	Educational level	Employment (paid)	Children	Living situation	Type of violence and abuse
226	43	Heterosexual	Dutch	Dutch+Indonesian	None or atheism	Higher vocational education	Yes	Yes	With children	Physical, psychological, and economic
290	46	Heterosexual	Dutch	Dutch+French	None or atheism	Higher vocational education	No	Yes	With children	Psychological and economic
322	38	Heterosexual	United States	Dutch+United States	None or atheism	Vocational education	Yes	Yes	With children	Physical, psychological, and economic
422	26	Bisexual	Dutch	Dutch	None or atheism	Higher vocational education	No	No	Alone	Physical, psychological, sexual, and economic
431^a^	48	Heterosexual	Dutch	Dutch	None or atheism	Higher vocational education	Yes	No	Alone	Physical and psychological
457	49	Heterosexual	Dutch	Dutch	None or atheism	Vocational education	No	Yes	With children	Psychological
501	39	Heterosexual	Dutch	Dutch	Christianity	Higher vocational education	No	Yes	With children	Physical, psychological, sexual, and economic
545	47	Bisexual	Dutch	Dutch	None or atheism	University	Yes	Yes	With children	Psychological
629	50	Heterosexual	Dutch	Dutch	None or atheism	Vocational education	Yes	Yes	Alone	Physical and psychological
647	50	Heterosexual	Dutch	Dutch	Christianity+Hinduism	University	No	Yes	With partner and children	Physical, psychological, and sexual

^a^This participant dropped out during the randomized controlled trial but did participate in the process evaluation interviews.

### Use and Ease of Use

For the control group (n=99), the database with help options was the most popular (Multimedia Appendix 2). This differed from the intervention group (n=99), which visited the chat and forum the most (chat: 308 times by 36/99, 36.4% of the women; forum: 601 times by 27/99, 27.3% of the women; Multimedia Appendix 2). However, only a small number of women actively posted one or more messages (chat: 20/99, 20.2%; forum: 2/99, 2%). In the forum, a participant posted a question on the study, and another posted a message asking for advice regarding death threats from her partner, which a community manager (CM; a mental health care professional that manages the platform) responded to. Third-party stories of survivors taken from web-based platforms and magazines and posted by the CMs were the most accessed features on the forum. Furthermore, transcripts from the themed chats were shared on the forum, and these were frequently read as well. The organized themed chats on psychological violence and abuse were the most popular (Multimedia Appendix 3). In the *live* themed chats, participants, survivor professionals (SPs; survivors who received training on how to use personal experiences in supporting other survivors or DVA and IPVA professionals who had personal survivor experience), professionals, and CMs discussed experiences, exchanged advice and tips, and provided encouragement and support:

I’m staying now because it’s been going well for a month.

Participant 429

He’s playing upon your hope and empathy.

SP

You always hope it’ll be better again. I’ve buried my head in the sand for a long time as well.

Anonymized participant

Yeah, I do constantly remain hopeful. But my boyfriend says that I have no empathy haha!

Participant 429

........................

You keep holding on because you get enough ‘good’ moments still. Unfortunately, those moments are part of the game.

SP

Yeah, that’s right but sometimes I also hear that we complement each other so well. I have the patience of a saint, he doesn’t. And sometimes he’s really grateful for that. It’s so contradictory sometimes. One moment everything’s peaches and cream and after that it suddenly explodes.

Participant 429

........................

I admire that you took these steps. I hope I will be able to do so as well.

Participant 429

I have faith in you [Participant 429]

SP

I think it’s really good that you have a lot of insight already! [Participant 429]

CM

With regard to the relatively limited use of the chat, the interviewees provided some explanations: if it is not very active, it is not encouraging to start a conversation, and if a few women are already having a conversation, other women may feel uncomfortable joining them. In addition, some may simply not feel the need to use the chat; a few interviewees said that it could even be triggering instead of helpful. Others can find it difficult to disclose their own experiences or dislike having to type their story. A woman said that she would like video calling in small groups to have a sense of more real contact that is still very accessible. In total, 20% (2/10) of the interviewees said that it was easier for them to talk to a friend or professional than to use the chat. Some interview participants faced obstacles, such as technological difficulties, not being able to find the chat on the website, or the timing of the themed chats, that could get in the way of their children’s bedtime or interfered with their abusive partner being home:

I thought that [themed chats] was very good...However, what I noticed was because I have a child, that the themed chat is often around 19:00 or 20:00. But around that time many parents put their children to bed, so that’s quite impractical...I think also in the evening, you know, when you’re in an abusive relationship and the partner’s at home, well then you’re not going on the chat.

Participant 290

There were similar explanations for the passive forum use, and interviewees said that the forum was less visible for them than the chat. They were happy to learn that they could read the previous themed chats on the forum as well; apparently, that was not always clear even though this was stated at the end of every themed chat and in the invitation emails for the themed chats as well.

Regarding ease of use, many of the questionnaire participants and some of the interview participants reported that they did experience some obstacles. They attributed this to a lack of a clear overview of where they could find certain items on the website. A more intuitive and immediate structure might be needed, especially for women who are in a very stressful situation that causes problems with processing information. However, other participants did not identify this issue and found the website very accessible and easy to navigate. Reactions to use on a mobile device were mixed, with some participants extremely satisfied and others preferring a big screen for viewing. A few women also experienced log-in problems, but these were always solved quickly:

Sometimes I found it difficult to use the website, to find certain things and to understand how to use it. That took a while to master. I think it’s also a matter of clicking through the website. You should actually go through all the steps but I think that most people are in survival mode and can’t go through all themes. It’s actually very helpful to go through all the steps because it increases your awareness.

Participant 322

Surprisingly, a few interviewees stated that they thought that the intervention had changed over time, although in fact nothing was modified during the RCT period except the constant updates in the dynamic sections, such as chat, forum, “News and more,” and the help database. This also reinforces the assumption that the perception of the website and its ease of use and accessibility might differ depending on the current situation of the user:

I can’t remember exactly when I’ve last seen the website but I have the impression that it’s really changed in the meantime. Yesterday I looked at the mobile website and I think it’s really good because it also works well on a mobile phone and that’s not always the case for websites. I think the design is great and super clear.

Participant 545

### Safety

Most survey participants (39/43, 90.7%; control and intervention groups combined) and all interview participants felt that the intervention was safe to use. Many participants mentioned the safety measures—escape button, a pop-up reminding to call 112 (the Dutch emergency telephone number) when in immediate danger, safety instructions, and contact emails sent under the title “Menstrual calendar.” Anonymity through being able to use a nickname was important to them, and they felt that their privacy was ensured by an independent, trustworthy party (university) that provided a shielded space. Knowing that there were fellow survivors who used SAFE also contributed to their sense of safety. Furthermore, some participants discussed circumstantial reasons for why they felt they could use the intervention safely, such as them not living with their abusive partner anymore or thinking that their partner did not know about the intervention (Multimedia Appendix 4).

However, a few participants from the survey and interview groups expressed worries about the security of their personal data and data being reported to other people or authorities. An interviewee thought that the escape button could also scare off some women who might feel that their situation is not severe enough and that they do not belong in the target group:

If I was still with my ex when I discovered this website then I would’ve been very happy with the escape button. It’s very good that you’ve thought about that but then I also think...Jeez, that’s actually really intense. And maybe that can also, especially for someone who’s in such a relationship, in a way be a mirror to show that the situation is that unsafe.

Participant 422

And the way you e-mail [ie, using “menstrual calendar” as e-mail subject], that’s also fantastic. I can imagine it’s necessary. I dealt with someone who controlled me in various ways, also digitally, so this really is a safe way to do it.

Participant 322

### Level of Satisfaction and Helpfulness

Within the survey group, participants in the intervention study arm graded the intervention significantly higher than those in the control study arm (mean 7.82, SD 1.859 and mean 6.07, SD 0.951, respectively; *P*<.001). Most participants from the intervention group (Multimedia Appendix 4) and the interview group said that the website was clear and helpful. They appreciated the information and help options provided and the level of safety and privacy:

If I’d had SAFE when I was still in that violent relationship it would’ve helped me tremendously.

Survey participant 549; intervention group

The ways in which the intervention was helpful were discussed in the interview group. Most women felt that SAFE was helpful in promoting awareness, self-reflection, acknowledgment, and processing and in providing support. Many said that it helped them realize that they were not alone in experiencing IPVA, especially because of the stories and short videos from survivors. Although not all interviewees felt that the intervention influenced their mental health, for example, in reducing stress, it did seem to help them be motivated to take action and feel empowered. The questionnaires, which were technically a research component instead of an intervention component, turned out to be helpful as well. A few interview participants said that they provided them with useful new insights:

Well I do think that it [SAFE] brought some peace just because you’re listened to. It also played a role in help seeking, I learned to acknowledge that it’s okay to accept help. I think that started with SAFE because you open up via the website first and then you can do the same with professional help. It’s hard to talk about what happened but I think SAFE did help with this.

Participant 647

I did regularly look if there was something I could use, just to deal with it in a constructive manner. So not just feeling those negative emotions but to also work on a solution. So indirectly you do provide a sort of light at the end of the tunnel...It helps when there’s a place where you...yeah, fellow survivors or where you can share your story...even if it’s just looking for information it can help to lower stress.

Participant 226

Regarding SAFE’s role in help seeking, some interviewees stated that they did not need help from other resources at that time or that professional help was already involved. Others did not feel that the intervention immediately helped them in contacting professional help, but some said that it did help in navigating the professional help realm and how to deal with professionals and in providing ideas that they could discuss with professionals:

...it’s really that low-threshold approach. To just be able to pour your heart out and to see if I can create a road map for myself to get out of this, that’s really great. Yeah, very important.

Participant 226

The intervention seemed to play a role in breaking the taboo around IPVA and making it a topic of discussion as well according to many interview participants. One woman said that SAFE encouraged her to talk about her situation with people in her community. Many interviewees wanted to share their experiences with other survivors. The help options, including options for contact with fellow survivors, and the contact options (forum and [themed] chat) partially provided a solution for this need:

...I started talking about it with people in my environment and they said they didn’t know it was this bad already...Many people didn’t believe me and my ex-partner comes from a closed community. So you didn’t talk about this with people outside the community...But now I think “suit yourself”...and I noticed that I found something that gave me a push and helped me to start talking about it.

Participant 647

Furthermore, interviewees appreciated the layout, although one woman suggested a calmer design, and the option to tailor the intervention to their own needs as there was no prescribed order for using the intervention.

### Points of Improvement

Some participants from the survey group (control arm and intervention arm combined) and the interview group reported a lack of clarity and overview. They could not always find what they were looking for, and some said that there were so many help options that it could be overwhelming. It would help if the information was tailored to specific IPVA situations and dynamics. In addition, a few interviewees mentioned tips for functionalities that were already present but that were apparently not visible or accessible enough, such as filter options in the help database and the forum.

Furthermore, the control group specifically mentioned the lack of interactive components and contact options such as a forum or chat and stories from survivors as these were only available to the intervention group. The intervention group reported the absence of certain types of information that they felt should be part of the intervention, for example, information on divorce and children and on why a partner becomes abusive (Multimedia Appendix 4). In addition, the interview group expressed a need for more information on legal affairs, professional help trajectories, digital, and technological IPVA (eg, stalking and intimidating via social media, hacking, and tracking):

It didn’t help me much. The information was already known to me but I mostly sought contact with fellow survivors. I didn’t find that.

Survey participant 641; control group

In total, 20% (2/10) of the interview participants stated that they had some doubts about whether they were part of SAFE’s target group as the home page seemed to focus on more severe violence, and women who did not have that experience may feel that their situation was not severe enough. One interviewee also said that she was looking for information and support for the phase after leaving their abusive partner, whereas the intervention seemed mainly focused on women who were still in the abusive relationship.

Finally, a few interviewees provided some other points for improvement and tips, for example, availability in multiple languages, webinars with more in-depth information, encouraging women to take good care of themselves, and a button on the website that immediately contacts emergency services and sends their location.

## Discussion

In the context of the Dutch SAFE eHealth intervention for women who experience IPVA, we investigated its impact [30] and the structural requirements of such a web-based platform to maximize its user-friendliness and the impact of the intervention. Herein, we present the results of the secondary aim.

### Principal Findings and Comparison With Prior Work

Consistent with findings from other studies, we found that participants rated the intervention in positive terms and found it helpful, for example, in awareness and feeling supported [12,13,16-18,35,36], with a more significant impact in the intervention study arm [17]. The difference between study arms probably arose because this group received a more elaborate and interactive version, and they spent significantly more time on it [30]. Participants also expressed points for improvement, such as a need for certain types of (additional) information on, for example, digital IPVA and legal affairs. Furthermore, although most found the intervention safe and easy to use [12,13,16,17,37], some also experienced difficulties navigating the website and reported a lack of clarity and overview.

In developing these types of interventions, it seems important to provide survivors’ stories, for example, through videos and third-party stories, and contact options such as a chat or forum. However, making these features available does not necessarily translate into their active use. Nevertheless, passive use, such as solely reading the posts of other users, could also be a valuable source of support for some IPVA survivors [13,18,19,38,39]. Some women in this study expressed a need for more interactive contact, but they faced certain obstacles at various levels: (1) practical (eg, timing of the themed chats and not knowing where to find the chat or forum on the platform), (2) personal (eg, no need for a chat or forum and not wanting to share their own experiences), and (3) social (eg, preference for in-person contact and hesitancy to start a conversation or join an ongoing conversation). Although the chat and forum were only accessible to participants, who could remain anonymous, and in a closed environment, worries about their privacy and safety (eg, because of fear of their partner or ex-partner, or knowing that content shared would become part of scientific research) could have been a barrier as well. The combination of personal needs, preferences, and disclosure motives influences how and why women use these interactive components and what they gain from using them [40]. To stimulate active use of these features, the chat and forum should be more visible, and 24/7 availability should be emphasized, or alternatively, access limitations should be clearly stated. The presence of a CM should also be clearly communicated. Their presence at specific times throughout the day or week could also encourage use by reluctant participants. Furthermore, the monthly themed chats with a professional or SP could be offered at different times to increase reach. As women may have privacy concerns even while remaining anonymous, clear data management information is essential. Users should be advised on how to browse safely (eg, not sharing identifiable personal details) and how their privacy and data safety are ensured [35,41-43].

According to Rempel et al [44], eHealth interventions for IPVA survivors mainly focus on safety planning and leaving the abusive partner, whereas there is a lack of support for women who have already left the abusive partner. Our intervention was developed primarily for women who recently experienced IPVA, and 52.5% (104/198) of the participants reported an incident within the week of registering for SAFE and in our RCT [30]. Thus, in addition to reaching our defined target group, we included women in other stages of the process, for example, moving on after ending an abusive relationship. In line with this, some participants reported a need for more information and advice about the period after leaving an abuser. This supports a broader approach for interventions, for example, by adding specific information tailored to the postrelational stage [44,45]. To immediately direct users toward the help they need, their current relationship status might be used as a screening question upon access.

Taken together, eHealth interventions can offer diversified help to survivors of IPVA in different phases. Although web-based means and interventions could never and should never replace face-to-face care, they stand out because of their unique characteristics that can be a valuable addition to regular help, such as anonymity, low threshold, flexibility, and client autonomy. They can be a stepping stone to care, a bridge during a waiting-list period, an additional form of support simultaneously or alternately to face-to-face care, or a form of aftercare. Web-based help has the ability to reach a larger group of survivors and can be used to meet certain needs, for example, contact with fellow survivors, the option to reread information or further delve into certain topics, and autonomy in the professional help trajectory and emotional and psychological processing [12,13,16-18,25,36,37,46].

Looking at the results of this qualitative evaluation, the SAFE eHealth intervention had a positive impact on most of the female IPVA survivors. We did not find significant between-group differences over time for self-efficacy, depression, and anxiety upon quantitative evaluation [30]; however, this qualitative analysis highlights other ways in which the participants benefited from the intervention. Our results highlight the need for multilayered analyses applying a mixed methodology to assess the intervention’s effectiveness and support in context [47].

### Strengths and Limitations

A strength of this study is the various qualitative data and methods used to evaluate the SAFE intervention, complementing the quantitative evaluation [30]. Furthermore, this qualitative evaluation included women who experienced various types of IPVA. This study population is also quite representative of the general Dutch population in terms of diversity in sexual orientation. A total of 80% (8/10; interview group) to 91% (59/65; survey group) of our sample reported being heterosexual, compared with approximately 95% in the general Dutch population [48]. This is also the case for cultural background—88% (57/65; survey group) to 90% (9/10; interview group) of our sample was born in the Netherlands, and 91% (59/65; survey group) to 100% (10/10; interview group) identified (partially) as Dutch. In the Netherlands, 93.2% of the general population has Dutch nationality [49].

Some limitations should also be considered when attempting to generalize our findings. First, we had relatively small sample sizes for the survey (n=65) and interview (n=10) data; thus, the outcomes may not reflect the experiences and views of the entire study sample (N=198). Furthermore, for the interviews, we did contact all women from the intervention study arm who agreed to being approached for follow-up research (n=82), but only 12% (10/82) consented to participate in the interview study. Owing to the small sample size, we report a lack of diversity in terms of educational level. We did not include participants from the control study arm in the interviews as they received a less extensive version of the intervention, and we wanted to limit their participation in this study. We deemed this to be a more ethical balance between what we provided for this group and the burden of participating in a scientific study. However, they did have the opportunity to express their opinions in the open questions of the surveys. Furthermore, we did not include these participants as they did not obtain access to the extended intervention and would have probably mostly requested more interactive elements, as identified in our preparatory interviews, which was confirmed by this group’s responses to the open questions in the surveys [18].

Second, the intervention was solely available in Dutch at the time of the study. Therefore, we did not reach women who did not comprehend (sufficient) Dutch but who may be in more susceptible positions regarding the risk of experiencing IPVA [50]. Currently, SAFE is partially translated into Arabic and English, and we have conducted a study among Arabic-speaking women with a migrant background in the Netherlands [51], which further confirmed the need for cultural sensitivity and availability in multiple languages [11,24,52].

Finally, as many but not all women in the Netherlands are sufficiently (digitally) literate, this intervention may not have been accessible to all women who experience IPVA even if they do proficiently comprehend Dutch [18].

### Implications

This qualitative evaluation of the SAFE eHealth intervention provides more elaborate and in-depth data on users’ experiences and views, complementing our quantitative evaluation [30]. We showed that a considerable proportion of the participants who took part in the SAFE study used this intervention and evaluated it in positive terms. Although the views of participants who did not fill out follow-up surveys are unknown, the study shows that there is a demand for the intervention and that it can meet certain needs of IPVA survivors. This qualitative study provides important insights for the ongoing development of web-based interventions for IPVA survivors. The assessment of these eHealth interventions should not only focus on outcomes from an RCT and on quantitative measures, but it should also include complementary qualitative data on the users’ experiences and views [47]. Indeed, this study enriches the quantitative evaluation of the SAFE intervention [30] and is important in creating a more nuanced, better-informed, real-world picture of its effects and meaning for the women who used it. Furthermore, it provides detailed insight into potential improvements for further development of the SAFE intervention.

Web-based interventions for IPVA survivors can be easily found on the web and implemented broadly without consuming extensive resources [30]. This study shows that women in different IPVA situations and stages could independently use this intervention also before, during, or after receiving different types of professional help. Web-based support cannot replace regular (face-to-face) care and support, but it has its own advantages, such as anonymity and flexibility; it can enable women to find survivors’ stories and share their own; and it can guide them to regular care and complement it. Both women and professionals seem to be interested in the possibilities of eHealth and combining it with regular care—blended care [11,15,36,53]. This potential can only be demonstrated by a more elaborate and varied assessment of these interventions. Future studies should not only focus on optimizing eHealth interventions, including options for fellow survivor contact, but they should also investigate how web-based tools and face-to-face professional help can complement each other and improve support for IPVA survivors.

## Appendix

Multimedia Appendix 1 Questions from the interview guide.

Multimedia Appendix 2 Images from the most preferred components of the SAFE intervention.

Multimedia Appendix 3 Chat and forum data.

Multimedia Appendix 4 Feedback on SAFE intervention from the questionnaires.

Multimedia Appendix 5 CONSORT-eHEALTH checklist (V 1.6.1).

## Abbreviations

CM: community manager
DVA: domestic violence and abuse
GCQ: General Characteristics Questionnaire
IPVA: intimate partner violence and abuse
RCT: randomized controlled trial
SP: survivor professional
WEQ: Web Evaluation Questionnaire

## Data Availability

The data sets generated and analyzed during this study are available upon reasonable request from the Dutch National Centre of Expertise and Repository for Research Data (DANS) repository [54].
