# Supplementary material for: Key Factors in Helpfulness and Use of the SAFE Intervention for Women Experiencing Intimate Partner Violence and Abuse: Qualitative Outcomes From a Randomized Controlled Trial and Process Evaluation
Source: J Med Internet Res. 2023 Aug 21;25:e42647. doi: 10.2196/42647 (PMC10477920; doi:10.2196/42647)
Supplement: Multimedia Appendix 1 [file jmir_v25i1e42647_app1.docx]

**Multimedia appendix 1. Questions from the interview guide.**

The original interview guide is in Dutch and the questions were translated into English for the purpose of sharing this study with a larger audience.

**General**

1. Can you tell something about yourself? (Activities in daily life such as work or study; household / family composition; your own youth etc.)

2. Would you want to tell something about the nature of the violence or abuse that you have experienced?

**Findability and awareness**

3. How did you find the SAFE website?

**Usage**

4. Do you feel that, regarding your personal situation, you had sufficient possibilities to access the website? (For example, regarding mental load, stress, danger.)

5. Do / did you find it easy to use the website?

6. How did you create a safe environment to use the website? And are you confident that SAFE is safe to use?

**Content**

7. What do you think of the content of SAFE? So, about the information you can find there etc.

8. Which components of the website were good or valuable according to you? And why?

9. Which components of the website were not good or were missing according to you? And why?

**Tips / points for improvement**

10. Do you have tips for us to further improve SAFE?

**Impact**

11. What did you expect from SAFE when you first started using it?

12. Do you feel like SAFE has helped or supported you in some way? And how, and why?

13. Do you feel like SAFE has had an impact on your mental health? For example, in feeling comfortable in your own skin or having less stress now you have used / are using the website?

14. Do you feel like SAFE has helped you in decision making? For example, in reaching out to professional help?

15. How do you think SAFE could help other women? In what setting could it offer support, and in what setting would it not be suitable?

**End**

16. Do you have other remarks or ideas that you would like to discuss with us, regarding SAFE or the study?

17. Do you have any questions about the interview or about SAFE?
