# Supplementary material for: Key Factors in Helpfulness and Use of the SAFE Intervention for Women Experiencing Intimate Partner Violence and Abuse: Qualitative Outcomes From a Randomized Controlled Trial and Process Evaluation
Source: J Med Internet Res. 2023 Aug 21;25:e42647. doi: 10.2196/42647 (PMC10477920; doi:10.2196/42647)

**Multimedia appendix 2. Images from the most preferred components of the SAFE intervention.**

The intervention study arm used the chat and forum the most, this component was only available to the intervention study arm. This is an image that shows part of the forum. The blue text balloons on the right can be clicked on to access the chat. The green envelope can be clicked on to send a personal message to the Community Managers.


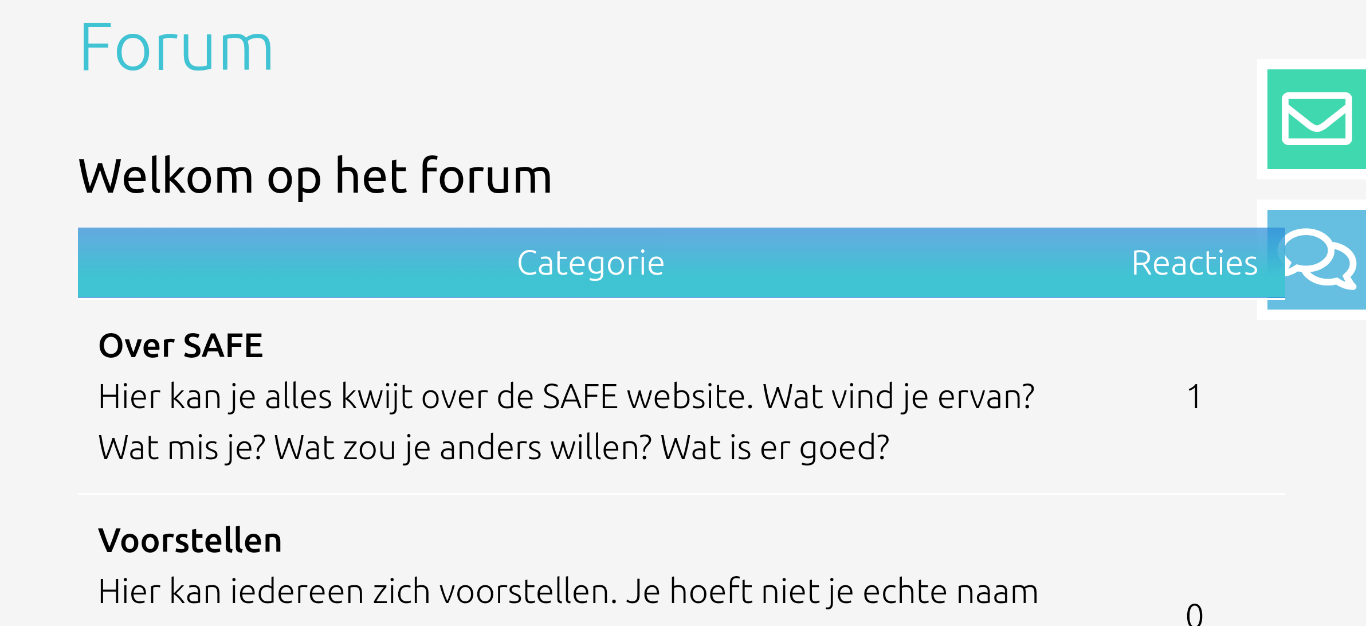


The control study arm used the database with help options the most, this component was available to both study arms. This is an image that shows part of the database.


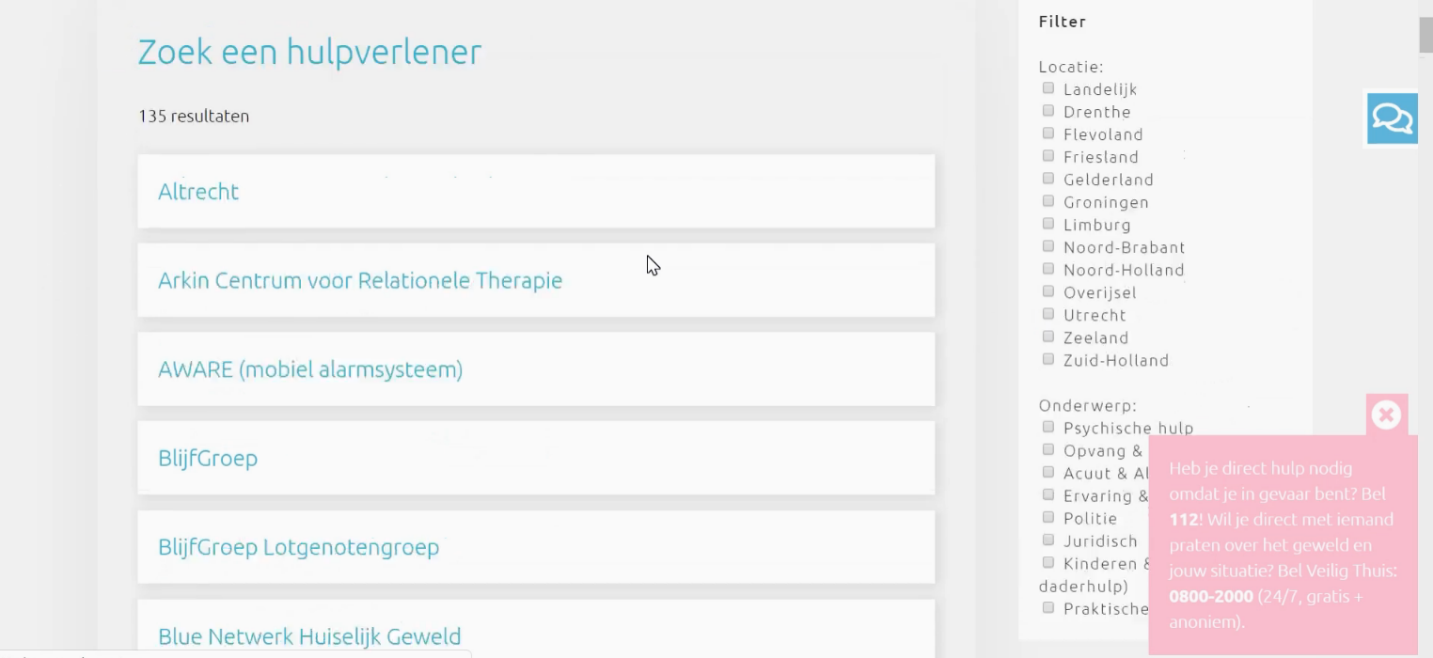

Supplement: Multimedia Appendix 2 [file jmir_v25i1e42647_app2.docx]
