# Supplementary material for: Key Factors in Helpfulness and Use of the SAFE Intervention for Women Experiencing Intimate Partner Violence and Abuse: Qualitative Outcomes From a Randomized Controlled Trial and Process Evaluation
Source: J Med Internet Res. 2023 Aug 21;25:e42647. doi: 10.2196/42647 (PMC10477920; doi:10.2196/42647)
Supplement: Multimedia Appendix 3 [file jmir_v25i1e42647_app3.docx]

**Multimedia appendix 3. Chat and forum data.**

| **Chat** | **Forum** |
| --- | --- |
| - Total amount of visits: **308** | - Total amount of visits: **601** |
| - Amount of unique visitors: **36** | - Amount of unique visitors: **27** |
| - Total amount of themed chats: **24 (monthly)** | - Total amount of topics: **8** |
| - Total amount of themes: **6^a^** | - Total amount of threads: **47** |
| - Total amount of themed chats with active participants: **11** | - Amount of active participants: **2** |
| - Amount of unique active participants within themed chats: **20** | - Most read topic: **survivors’ stories** |
| - Most popular themed chat: **psychological violence and abuse** |  |
| - Amount of unique active participants outside themed chats: **10** |  |

^a^Themes: Emotions and physical complaints; Psychological violence and abuse; Help seeking; IPVA and the nuclear family / the children; Your life after IPVA; Support from police and justice.
