# Supplementary material for: Key Factors in Helpfulness and Use of the SAFE Intervention for Women Experiencing Intimate Partner Violence and Abuse: Qualitative Outcomes From a Randomized Controlled Trial and Process Evaluation
Source: J Med Internet Res. 2023 Aug 21;25:e42647. doi: 10.2196/42647 (PMC10477920; doi:10.2196/42647)
Supplement: Multimedia Appendix 4 [file jmir_v25i1e42647_app4.docx]

**Multimedia appendix 4.** **Feedback on SAFE intervention from questionnaires.**

|  | **Intervention arm - Good points** | **Intervention arm - Points for improvement** | **Intervention arm - Other** |
| --- | --- | --- | --- |
| **GCQ - Experience with SAFE (unique N=22)** | - Nice to be heard. | - Login problems. | - I didn’t have time to look at it yet. |
|  | - Good to be included in research. | - More interaction / chats amongst survivors. | - I didn’t need this type of help anymore. |
|  | - The website provides insight on IVPA and can support women in making the decision to leave a violent relationship. | - When you’ve decided to leave the violent relationship you do need real help from real people. *(The researchers interpret this as a call for connecting online help to offline help.)* | - I don’t use it much anymore. |
|  | - A lot of information and help options. | - Some more feedback would be nice. |  |
|  | - The e-mails (reminders) help to reflect on how it’s going. | - More clarity (overview). |  |
|  | - The questionnaires. | - For the themed chats in the evening: take bed times of children into account so it’s easier for mothers to join the chat. |  |
|  | - Good website and initiative, low threshold, it feels really good and safe. | - It didn’t help / support me yet. |  |
|  | - It’s important that there’s a place with expertise on IPVA that offers support. It’s also confronting. | - Phone numbers from organizations need to be updated. |  |
|  | - It helps me realize that I’m not the only one, that IPVA has various forms, and what the facts of my situation are. It helps me to prevent that I’m downplaying what’s going on. | - More information on help regarding the situation after a (complex) divorce. |  |
|  | - Acknowledgement. | - More direct contact with professionals outside office hours. |  |
|  | - (Automated) e-mails sent under the name ‘Menstrual calendar’. | - I’m missing acknowledgement for my situation because it’s unusual and very complex. |  |
|  | - If I’d had SAFE when I was still in that violent relationship it would’ve helped me tremendously. | - I would like to share my story and that others can comment on it without direct contact. |  |
|  | - I only just realized how good it feels to talk about this because of using this. | - More stories from survivors. |  |
|  |  | - More information on the period after leaving the violent relationship and dealing with PTSD, trauma, and anxiety. |  |
|  |  | - Make it more suitable for various situations and relationship dynamics. |  |
| **WEQ - Points for improvement^a^ (unique N=9)** | N/A | - Nothing: 55,6% (N=5) | N/A |
|  |  | - Less youthful: 11,1% (N=1) |  |
|  |  | - Less old: 0% (N=0) |  |
|  |  | - Too much color: 0% (N=0) |  |
|  |  | - More colors and photos: 0% (N=0) |  |
|  |  | - Different logo: 0% (N=0) |  |
|  |  | - Different font: 0% (N=0) |  |
|  |  | - Menu: 0% (N=0) |  |
|  |  | - Other, namely: 33,3% (N=3)^b^ |  |
| **WEQ – Explanation for grade given (unique N=15)** | - Clear (overview), innovative, helpful. | - Missing information on why partners can be abusive. | - Haven’t had time yet to discover everything. / I’m not familiar with the website yet, I need more time to dive into it. |
|  | - Handles privacy and safety well. | - Can be more clear (overview). |  |
|  | - Good information (about what steps you can take) and sufficient amount of information. | - There are always some points for improvement. |  |
|  | - It’s nicely set up and it’ll probably expand. | - More videoclips and stories from survivors. |  |
|  | - Does what it says it does. | - Focuses on extreme cases, more preventative information could be added. |  |
|  | - Good initiative to help women. | - I don’t understand the website. |  |
|  | - Really good website. | - More (international) scientific information could be added. |  |
|  | - User friendly. |  |  |
| **WEQ – Explanation for feeling safe (unique N=15)** | - Privacy is ensured. | - It’s safe because it’s not interactive, no one talks to me. *(Interpreted by researchers as general point for improvement: more interaction.)* | - I’m not living together with my partner anymore. |
|  | - Website has a friendly appearance. | - I’m not sure if my personal data and the things I do on the website are very well secured. | - I didn’t know about this so I think my partner doesn’t either. / My partner doesn’t know about this website. |
|  | - Escape button and immediately going to Google. | - I’m still afraid other people and official authorities can read and report it. |  |
|  | - I can click away when I need to. |  |  |
|  | - It’s made for a group that finds safety very important. |  |  |
|  | - Pop-up with reminder to call 112 when in immediate danger. |  |  |
|  | - (Automated) e-mails sent under the name ‘Menstrual calendar’. |  |  |
|  | - It feels trustful. |  |  |
|  | - It’s shielded. |  |  |
|  | - Anonymity. |  |  |
|  | - There are many safety measures. |  |  |
| **WEQ – Opinion about intervention (unique N=17)** | - Clear (overview), useful, nice, super, very good, handy, well organized. | - Missing recent articles and publications on this subject. | - I didn’t spend enough time on it yet. |
|  | - Good to have someone that help to think with me and check if I’m okay. | - I already knew half of the information that was provided. | - I’m not sure how I feel about it yet, I need more time to go through it. |
|  | - Interesting but also confronting. | - It’s still unclear to me but maybe this has to do with my situation and that I needed to create safety for my family first. |  |
|  | - Good something like this exists. / Good initiative. | - I expected some more scientific input but maybe that will happen later. |  |
|  | - A place where I’m aware that I’m not the only one and that there is help. Also, that it’s not my fault. | - I’m suspicious of professionals and authorities so I’m afraid of doing things, I feel lonely in dealing with my problems. |  |
|  | - The information was useful to asses and reflect on my situation. |  |  |
|  | - Good guidance, good help. |  |  |
|  | - A (almost) recent overview of help options. |  |  |
|  | - Tailored to safety needs with regard to an abusive partner looking through your phone or looking over your shoulder. I was afraid of taking action but SAFE really helped me. |  |  |
| **WEQ – Missing in intervention (unique N=16)** | - Nothing, not applicable. | - Information on how to deal with professionals who work from the standpoint that there’s equality between the partners and in that way provide wrong or bad help or even make it dangerous. | - I don’t know. |
|  |  | - Information on motivations for a partner to become coercive, abusive or violent, why does this happen? | - Myself. *(Unknown for the researchers what this participant means exactly.)* |
|  |  | - More research and surveys to urge politicians and the government to take action in tackling violence against women. |  |
|  |  | - A quick link. *(Unknown for the researchers what this participant means exactly.)* |  |
|  |  | - Addresses for shelters and safe houses. |  |
|  |  | - Scientific evidence based knowledge, not only for SAFE but in general. Too little is being done and more awareness is needed. |  |
|  |  | - More information on plans to educate police officers and other professionals. |  |
|  |  | - Information on what happens with the children after a divorce. |  |
|  |  | - More stories from survivors. |  |
|  |  | - Chat with women who deal with the same problems. |  |
|  | **Control arm - Good points** | **Control arm - Points for improvement** | **Control arm - Other** |
| **GCQ - Experience with SAFE (unique N=23)** | - It’s safe and accessible. | - More articles and videoclips. | - I registered but haven’t used it yet. |
|  | - Escape button. / Easy to leave the website. | - Chat option. | - I haven’t used SAFE enough. |
|  | - Pop-up with reminder to call 112 when in immediate danger. | - Education on mechanisms in domestic violence. | - I only filled out the questionnaires but didn’t use the intervention. |
|  | - Useful and supportive messages on the SAFE Facebook page. | - More anonymous help options. | - I forgot about it. |
|  | - Good that SAFE remains contact via e-mails (reminders) and surveys. | - Login problems. / Problems with creating a new password. |  |
|  | - A lot of information and survivor stories. | - More information on IPVA, felt like it was only for research. |  |
|  | - Anonymity. | - I expected more from it, too little (new) information. |  |
|  | - The questionnaires. | - Everything, it doesn’t work and I want to delete my account. |  |
|  | - Good that it exists. / It gives me a good feeling to know this exists. / Fantastic initiative. | - (Automated) e-mails sent under the name ‘Menstrual calendar’ because I don’t menstruate so how would I explain it to my ex if he saw it? |  |
|  | - Support for women in all kinds of IPVA situations. | - In some cases it may be appropriate to discuss signs with professionals. |  |
|  | - SAFE is a good to checkpoint to see how it’s going. | - Via an IP-tracker even an anonymous reporter can be traced. |  |
|  | - Research on psychological violence, it’s supportive that the scientific community takes this seriously. | - It didn’t help me yet. |  |
|  | - Clear and comprehensible. | - Unclear (overview). / I don’t know where to begin. |  |
|  |  | - More tips on how to deal with IPVA. |  |
|  |  | - More support options / feeling supported. |  |
|  |  | - Contact with survivors more prominent / available. |  |
|  |  | - SAFE feels more like an obligation than something supportive at the moment. |  |
| **WEQ - Points for improvement^a^ (unique N=19)** | N/A | - Nothing: 42,1% (N=8) | N/A |
|  |  | - Less youthful: 0% (N=0) |  |
|  |  | - Less old: 0% (N=0) |  |
|  |  | - Too much color: 10,5% (N=2) |  |
|  |  | - More colors and photos: 10,5% (N=2) |  |
|  |  | - Different logo: 0% (N=0) |  |
|  |  | - Different font: 0% (N=0) |  |
|  |  | - Menu: 21,1% (N=4) |  |
|  |  | - Other, namely: 21,1% (N=4)^b^ |  |
| **WEQ – Explanation for grade given (unique N= N/A)** | N/A | N/A | N/A |
| **WEQ – Explanation for feeling safe (unique N= N/A)** | N/A | N/A | N/A |
| **WEQ – Opinion about intervention (unique N=28)** | - Useful information, help options and links. | - Lack of new and regional information. | - I don’t use it much anymore. / I use it less now. |
|  | - Sufficient amount of information. | - Didn’t suit to the type of help I need (legal, housing and shelter, divorce with children involved). | - I think it’s good but now I also started with professional help so I need it less. |
|  | - Clear (overview). | - Lack of clarity (overview), trouble finding things. | - I often forget it exists. |
|  | - Supportive. | - I miss direct steps I can take, I hoped to immediately have contact with someone and receive help but it’s just a collection of all kinds of information. |  |
|  | - Nice, fine, handy, very good initiative, good that this website exists. | - I notice that when I’m really in need, I don’t go to the website. |  |
|  | - Pleasant in usage and in contact. | - Little to offer, I don’t understand it so I don’t use it. |  |
|  | - Provides clear help options. | - I don’t know, I’ve only filled out a questionnaire and that’s it. / There are only questionnaires. |  |
|  |  | - Links (to events) could be updated more. |  |
|  |  | - A bit disappointing, it doesn’t help much. I did found a course, that’s it. |  |
|  |  | - Not really helpful. |  |
|  |  | - Not easy to use, I can’t change my password. |  |
| **WEQ – Missing in intervention (unique N= N/A)** | N/A | N/A | N/A |

^a^This question was only asked at the WEQ for one month and participants could check multiple answers, hence the total percentage can be above 100%. | ^b^Other, namely: intervention group = more mobile phone friendly, more stories from survivors, variation in short videos and stories from survivors; control group = information presented more clearly and easier to find, external links should open in a new tab, better content, a forum for fellow survivors.
